# Aseptol, the New Antiseptic

**Published:** 1885-03

**Authors:** 


### Aseptol, The New Antiseptic.

This new phenol compound has recently been brought to the notice of the profession under the name of ortho-oxyphenyl-sulphurous acid, a name which at a glance is intended to make clear its chemical composition. For greater convenience it has been decided to call it aseptol, a name derived from its property of completely destroying the lower forms of life.

Aseptol is said to possess properties which place it far in advance of anything hitherto offered for like purposes.

It is said to be highly germicidal, to be but feebly caustic, to be abundantly soluble, to possess but a slight, not disagreeable odor, and, so far as experiment has gone, to be far less toxic than carbolic acid. To this last body it stands related in a chemical as well as germicidal sense, and is applicable to all the uses to which it is put. Furthermore, owing to the greater solubility and the comparative innocuousness of the aseptol, it will find many uses where the other body, for obvious reasons, is wholly inapplicable.

Aseptol is described as being a viscid liquid of a slightly red color and of a specific gravity of 1.450; its odor suggests that of carbolic acid, but it is far more agreeable. It is suppled by Merck in a solution containing thirty-three and a third per cent. of the substance, and in this form it is eligible for immediate use or for dilution to any desired strength. From the report of Drs. Leroy and Von der Shriek, of Antwerp, who studied quite extensively its therapeutic applications, the following table of advantages is derived:

1. It is very soluble in water.
2. It is very slightly caustic.
3. It is free from irritating qualities, and may be applied for a long time to the skin, the eyes, the bladder, etc.
4. Finally from its slight toxicity, which permits its use internally in considerable doses, and also the application of it in concentrated solutions to diphtheritic pharyngitis, we anticipate for it a wide field of usefulness.

